# The impact of age and renal function on the pharmacokinetics and protein binding characteristics of fludarabine in paediatric and adult patients undergoing allogeneic haematopoietic stem cell transplantation conditioning

**DOI:** 10.1007/s00228-024-03751-0

**Published:** 2024-09-19

**Authors:** Christa E. Nath, Sebastian P. A. Rosser, Kiran K. Nath, Jason Chung, Stephen Larsen, John Gibson, Melissa Gabriel, Peter J. Shaw, Steven J. Keogh

**Affiliations:** 1 Department of Biochemistry, The Children’s Hospital at Westmead, Westmead, NSW 2145 Australia; 2 The Cancer Centre for Children, The Children’s Hospital at Westmead, Westmead, NSW 2145 Australia; 3 Royal Prince Alfred Hospital, Camperdown, NSW 2006 Australia; 4 Sydney Pharmacy School, University of Sydney, Camperdown, NSW 2006 Australia; 5 The Children’s Hospital at Westmead Clinical School, University of Sydney, Westmead, NSW 2145 Australia; 6 School of Psychology, Western Sydney University, Kingswood, NSW 2747 Australia

**Keywords:** Fludarabine, Population pharmacokinetics, Protein binding

## Abstract

**Aim:**

To evaluate the population pharmacokinetics of unbound F-Ara-A (the circulating metabolite of fludarabine) in 211 patients (age range, 0.1–63.4 years) undergoing allogeneic haematopoietic stem cell transplantation conditioning.

**Methods:**

Total (*n* = 2480) and unbound (*n* = 1403) F-Ara-A concentrations were measured in blood samples collected at timed intervals after fludarabine doses ranging from 10 to 50 mg/m^2^ and infused over 0.42–1.5 h. A three-compartment population pharmacokinetic model was developed based on unbound plasma concentrations and used to estimate F-Ara-A unbound pharmacokinetic parameters and fraction unbound (*fu*). A number of covariates, including glomerular filtration rate (GFR) and post-menstrual age (PMA), were evaluated for inclusion in the model.

**Results:**

The base population mean estimates ± relative standard error (%RSE) for unbound clearance from the central compartment (CLu) and inter-compartmental clearances (Q2u, Q3u) were 3.42 ± 3%, 6.54 ± 24% and 1.47 ± 16% L/h/70 kg, respectively. The population mean estimates (%RSE) for the unbound volume of distribution into the central (V1u) and peripheral compartments (V2u, V3u) were 9.65 ± 8%, 8.17 ± 9% and 16.4 ± 10% L/70 kg, respectively, and that for *fu* was 0.877 ± 1%. Covariate model development involved differentiating F-Ara-A CLu into non-renal (1.81 ± 9% L/h/70 kg) and renal components (1.02 ± 9%*GFR L/h/70 kg). A sigmoidal maturation factor was applied to renal CLu, with population mean estimates for the Hill exponent and PMA at 50% mature of 2.97 ± 4% and 69.1 ± 8% weeks, respectively.

**Conclusion:**

Patient age and GFR are predictors of unbound F-Ara-A CLu. This has the potential to impact dose requirements. Dose individualisation by target concentration intervention will be facilitated by this model once it is externally validated.

**Supplementary Information:**

The online version contains supplementary material available at 10.1007/s00228-024-03751-0.

## Introduction

Fludarabine (fludarabine phosphate) is a purine analogue and an immune suppressive antineoplastic agent that is widely used in allogeneic haematopoietic stem cell transplantation (HSCT) conditioning regimens [1, 2]. Combining an immune suppressive agent, such as fludarabine or cyclophosphamide with alkylating agents such as busulfan, melphalan or treosulfan, improves engraftment and durability of cells by altering the host’s immune system to “create room” for the donor cells [1, 2]. Whilst fludarabine has been incorporated into myeloablative HSCT conditioning regimens [3–5], the toxicity and efficacy profile of fludarabine makes it a critical component for a variety of reduced intensity HSCT regimens [1, 2]. This versatility extends the option of treatment with HSCT to vulnerable patients and those with specific diseases that do not require myeloablative HSCT. Optimisation of fludarabine dose has been raised as an important issue as it might impact on engraftment, relapse and the incidence of adverse effects [3].

Fludarabine is a prodrug that is rapidly de-phosphorylated to the primary metabolite in the systemic circulation, 9β-D-arabinosyl-2-fludarabine (F-Ara-A) [6]. F-Ara-A is then re-phosphorylated intracellularly to the active 9β-D-arabinofuranosyl-2-fluoroadenine triphosphate, which is responsible for the inhibition of DNA and RNA synthesis in tumour cells, leading to apoptosis [6]. Between 40 and 60% of the fludarabine dose has been recovered in the urine of patients in the form of F-Ara-A within 24 h from dose administration [7, 8], suggesting that renal excretion has an important role in F-Ara-A elimination. The importance of renal excretion as an elimination pathway has also been identified in a number of previous pharmacokinetic studies conducted in children [9–13] and adults [13–15]. Fludarabine dose reductions have been recommended that are dependent on the degree of renal impairment [16–18]. Other factors shown to be associated with F-Ara-A clearance in previous population pharmacokinetic studies include body weight [12], fat-free mass [10] and maturational factors [10].

Fludarabine has been shown to bind to human serum albumin [19]. Whilst protein binding of F-Ara-A has previously been examined in vitro, with minimal protein binding observed (approximately 1%) [8], there is limited in vivo data. Unbound F-Ara-A concentrations were measured as part of phase 1 pharmacokinetic evaluations for fludarabine doses ranging from 80 to 260 mg/m^2^ [15], but more recent data for current doses is lacking. Data on unbound F-Ara-A clearance is important for dose optimisation studies for targeting unbound AUC.

The aims of this study were to (1) examine the population pharmacokinetics of unbound F-Ara-A in paediatric and adult patients who received fludarabine as part of allogeneic HSCT conditioning; (2) evaluate the impact of covariates, including age and glomerular filtration rate (GFR), on the unbound pharmacokinetic parameters; and (3) compare the pharmacokinetic parameter estimates with those obtained in previously published studies.

## Materials and methods

### Patients and study design

A total of 211 patients aged between 0.1 and 63 years were recruited from two sites in New South Wales, Australia. The study was a prospective investigation on the pharmacokinetics of F-Ara-A in patients ranging from neonates to adults who were administered fludarabine as part of HSCT conditioning. Participants were recruited either to a multi-centre clinical trial entitled “Pharmacokinetics, pharmacodynamics and pharmacogenomics of busulfan and other agents used in blood or marrow transplantation”, registered with the Australian Clinical Trials Registry (Registration number: ACTRN12612000544875, registration date: 22/05/2012) or an earlier single-centre study “Pharmacokinetics of fludarabine des phosphate in children receiving fludarabine”. The study protocols were approved by the Sydney’s Children’s Hospital Network Human Research Ethics Committee, and written consent was obtained from parents/guardians of children and/or participants.

### Fludarabine dose administration

The patients in this study were treated according to specific BMT conditioning protocols which varied with respect to the total administered fludarabine dose and the number of days over which it was administered (range, 3 to 6 days). Fludarabine was infused over 0.42 to 1.5 h (median 0.5 h). The BMT conditioning protocols used combinations that contained busulfan in 155 transplants (73%) and treosulfan in 35 transplants (17%) (Table 1); in these protocols, fludarabine was administered immediately prior to the busulfan or treosulfan infusions. A surface area-based fludarabine dose (range, 10 to 50 mg/m^2^) was administered in 194 transplants, whilst a weight-based dose of 1 to 2 mg/kg was used in 18 transplants where the patients weighed < 11 kg. In two patients with GFR values of 41 and 30 mL/min/1.73 m^2^, respectively, the initial fludarabine dose was reduced by 50%. Real-time F-Ara-A pharmacokinetic assessment based on total F-Ara-A concentrations was conducted in two patients, a child with a GFR of 30 mL/min/1.73 m^2^ and a pre-term baby transplanted at term, and this led to further fludarabine dose reductions following a reduced initial dose.

**Table 1:** Clinical data of 211 patients who received fludarabine as part of allogeneic HSCT conditioning

**Characteristic**	
**Number of patients**	211
**Number of concentration observations (Total /unbound F-Ara-A)**	2480 / 1403
**Physiological characteristics, median (range)**	
Age, years	8.14 (0.11 – 63.4)
0 – 1 year (n)	29 (14%)
1- 7 years (n)	69 (33%)
7-12 years (n)	38 (18%)
12 – 20 years (n)	48 (22%)
> 20 years (n)	27 (12%)
Body weight, kg	24.4 (2.9 – 124.3)
Height, cm	124.3 (48 – 186)
Body surface area, m^2^	0.94 (0.20 – 2.47)
Glomerular filtration rate (mL/min/1.73 m^2^)	110.7 (30 – 300)
Glomerular filtration rate (mL/min)	60.7 (6 – 191)
Sex (n)	
Male	135 (64%)
Female	76 (36%)
**Individual condition (n)**	
Haematological malignancy	123 (58%)
acute myeloid leukaemia	56
acute lymphoblastic leukaemia	32
myelodysplasia syndrome	16
Non-Hodgkin Lymphoma	5
juvenile myelomonocytic leukaemia	3
Other	11
Inborn errors of immunity	47 (22%)
severe combined immunodeficiency	11
chronic granulomatous disease	7
Wiskott Aldrich syndrome	5
other primary immune deficiency diseases	24
Other genetic diseases	41 (20%)
mucopolysaccharidosis	17
aplastic anaemia	11
fanconi anaemia	2
mannosidosis	2
Juvenile osteopetrosis	2
Other	7
**Donor source (n)**	
Matched related donor	89 (42%)
Matched unrelated donor	120 (57%)
Mismatched related donor	1 (0.5%)
Autologous salvage	1 (0.5%)
**HSC source (n)**	
Bone marrow	72 (34%)
Peripheral blood	105 (50%)
Cord blood	31 (15%)
Bone marrow and cord	2 (1%)
**Conditioning regimen (n)**	
**Busulfan-based**	**155 (73%)**
Busulfan/fludarabine	63
Busulfan/fludarabine/melphalan	51
Busulfan/fludarabine/melphalan/thiotepa	1
Busulfan/fludarabine/thiotepa	15
Busulfan/fludarabine/clofarabine	3
Busulfan/fludarabine/cyclophosphamide	21
Busulfan/fludarabine/clofarabine/cyclophosphamide	1
**Treosulfan-based**	**35 (17%)**
Treosulfan/Fludarabine	16
Treosulfan/Fludarabine/Thiotepa	15
Treosulfan/Fludarabine/Thiotepa/etoposide	1
Treosulfan/Fludarabine/Melphalan	2
Treosulfan/fludarabine/cyclophosphamide	1
**Other**	**21 (10%)**
Fludarabine/thiotepa/melphalan	1
Fludarabine/cyclophosphamide	10
Fludarabine/cyclophosphamide/total body irradiation	4
Fludarabine/melphalan/total body irradiation	1
Fludarabine/thiotepa/cyclophosphamide/total body irradiation	3
Fludarabine/dexamethasone/total body irradiation	1
Fludarabine	1
**Immunosuppressive agents (n)**	
Alemtuzumab	33 (16%)
Anti-thymocyte globulin (ATG)	49 (23%)
None	129 (61%)
**Fludarabine dose (median, range)**	
Fludarabine daily dose (mg)	27 (5 – 100)
Fludarabine daily dose (mg/m^**2**^)	30.3 (9.65 – 51.5)
Fludarabine cumulative dose (mg/m^**2**^)	148.7 (23.8 – 248.8)
Fludarabine daily dose (mg/kg)	1.14 (0.29 – 2.63)
Fludarabine cumulative dose (mg/kg)	5.07 (0.85 – 9.94)
**Transplant centre (n)**	
The Children’s Hospital at Westmead	183 (87%)
Royal Prince Alfred Hospital	28 (13%)

### Blood sampling for F-Ara-A pharmacokinetic analysis

A series of five to eight blood samples were collected at timed intervals after the first, second, third, fourth and fifth fludarabine doses. In 155 patients who received busulfan, fludarabine was infused immediately prior to the busulfan infusion, with blood for fludarabine concentration assessment collected at the end of the fludarabine infusion then at 0 h, 1 h, 2 h, 4 h and 8 h after the busulfan infusion (1.5- to 3-h duration). Similarly, in 35 patients who received treosulfan, fludarabine was infused immediately prior to the treosulfan infusion (2-h duration), with blood collection at the end of the fludarabine infusion (then at 0 h, 0.5 h, 1 h, 3 h, 5 h, 7 h and 12 h after the treosulfan infusion). In those children who did not receive busulfan or treosulfan, blood sampling was at 0 h, 0.25 h, 0.5 h, 1 h, 3 h, 6 h and 12 h after the end of the fludarabine infusion. A total of 74 patients also had samples collected from between 12 and 24 h after the dose. Bloods were centrifuged for 10 min at room temperature at 3400 rpm within 60 min of collection and the plasma was stored at − 40 °C until analysis.

### F-Ara-A concentration measurement

The clinical pharmacokinetics of fludarabine was evaluated by studying the total and unbound concentrations of the primary metabolite, F-Ara-A. Detection and quantification of total and unbound F-Ara-A were carried out using a Shimadzu Nexera ultra-high-performance liquid chromatography (UHPLC) system fitted with two LC-30AD pumps, a SIL-30AC autosampler and a SPD-M30A photodiode array detector set at 262 nm. For determination of total plasma concentration of F-Ara-A, 100-µL plasma aliquots from patient samples and spiked standards (0.1, 0.2, 0.5, 0.75, 1, 2, 5 µg/mL) were deproteinized by addition of 25% trichloroacetic acid (50 µL) and water (150 µL). After vortex mixing and centrifuging at 13,000 rpm, a 20-µL aliquot of the supernatant was injected into the UHPLC system. For determination of unbound F-Ara-A concentration, 200-µL aliquots of patient plasma samples were deproteinized by rapid ultrafiltration with Amicon 0.5-mL ultracentrifugal filters (PN: UFC501096) that utilized regenerated cellulose with a molecular weight cut-off of 10,000 units. Calibration was against spiked standards (0.1–5 µg/mL) prepared in water, a similar aqueous matrix as plasma ultrafiltrate. HPLC separation was achieved using a Kinetex XB C18 2.9 µm 100 × 4.6 mm column (Phenomenex 00D-4496-E0) fitted with a C18 4.6 mm ultra security guard (Phenomenex: AJO-8768) using an isocratic mobile phase that consisted of 50 mM sodium phosphate buffer, pH 4, with 9% acetonitrile and 0.1% sodium octyl sulphonate (10 g/100 mL). Using a flow rate of 1 mL/min, the retention time of F-Ara-A was 4.3 min. The total F-Ara-A assay was linear from 0.1 to 5 µg/mL with acceptable precision (< 13% coefficient of variation, CV) and accuracy (bias < 6% deviation from the nominal concentration) for spiked plasma controls, with F-Ara-A concentrations of 0.2, 0.75, 1.5 and 5 µg/mL (*n* = 66). The limit of quantification (LOQ) for the total F-Ara-A assay was 0.1 µg/mL (CV = 20%, bias = 1%, *n* = 66). The unbound F-Ara-A assay was linear from 0.1 to 5 µg/mL with acceptable precision (CV < 15%) for the same spiked plasma controls (*n* = 55). A negative bias was observed, with estimates of 26%, 21%, 16% and 16% recorded for the 0.2, 0.75, 1.5 and 5 µg/mL concentrations, respectively. This may not reflect the true accuracy of the unbound F-Ara-A assay as protein binding may have occurred during the spiking process. The LOQ for the unbound F-Ara-A assay was also 0.1 µg/mL (CV = 13%, bias =  − 23%, *n* = 6).

### Pharmacokinetic data

The pharmacokinetic data that was collected included the date and time of fludarabine dose and the date and time of blood collections for F-Ara-A concentration determination. Since the measured F-Ara-A is different from the administered dose (fludarabine phosphate), an equivalent dose of F-Ara-A was used for modelling. This was calculated as the fludarabine dose multiplied by 0.78, which is equivalent to the ratio of F-Ara-A molecular weight (285 g/mol) to fludarabine phosphate molecular weight (365.2 g/mol).

Other data that was collected included patient or treatment-related factors that had the potential to impact the F-Ara-A pharmacokinetic parameters. Data on patient age, sex, weight and height was collected and allowed calculation of other parameters related to body size including body surface area (BSA), body mass index (BMI) and fat-free mass (FFM). BSA was calculated using the equation of Mosteller et al. [20]. FFM was calculated using published formulas for adults [21] and children [22]. Post-menstrual age (PMA, weeks) was calculated as postnatal age + gestational age (40 weeks in all patients except one pre-term infant for whom gestational age was 34.3 weeks). GFR was determined by measuring the plasma clearance of _43_Tc^99^–diethylenetriaminepentaacetic acid for 181 children and the Schwartz-Lyon equation [23] for two children. In adult patients, GFR was estimated using the Chronic Kidney Disease Epidemiology Collaboration (CKD-EPI) equation [24]. Other information that was collected included whether or not there was pre-existing liver impairment, HSCT conditioning (busulfan-based, treosulfan-based or other), diagnosis group (malignant, immune deficiency or genetic disease), pre HSCT albumin and total protein concentrations and concomitant medications during the period of fludarabine administration, including ganciclovir, allopurinol, clobazam, clonazepam, amphotericin B, fluconazole and serotherapy.

### Population pharmacokinetic modelling of total and unbound F-Ara-A concentrations

Population PK modelling was performed using nonlinear mixed effects modelling software (NONMEM, version 7.40) with the Perl-speaks-NONMEM library (version 4.9.0) [25] and Pirana (version 2.9.9) as the graphical user interface [26]. A first-order conditional estimation with interaction (FOCE-I) method was used for approximation. Graphical output and statistical analysis were performed using the ggplots2 [27] and ggstatsplot [28] packages implemented in R (version 4.2.3) and RStudio (version 2023.03.0 + 386) [29].

**Fig. 1:**
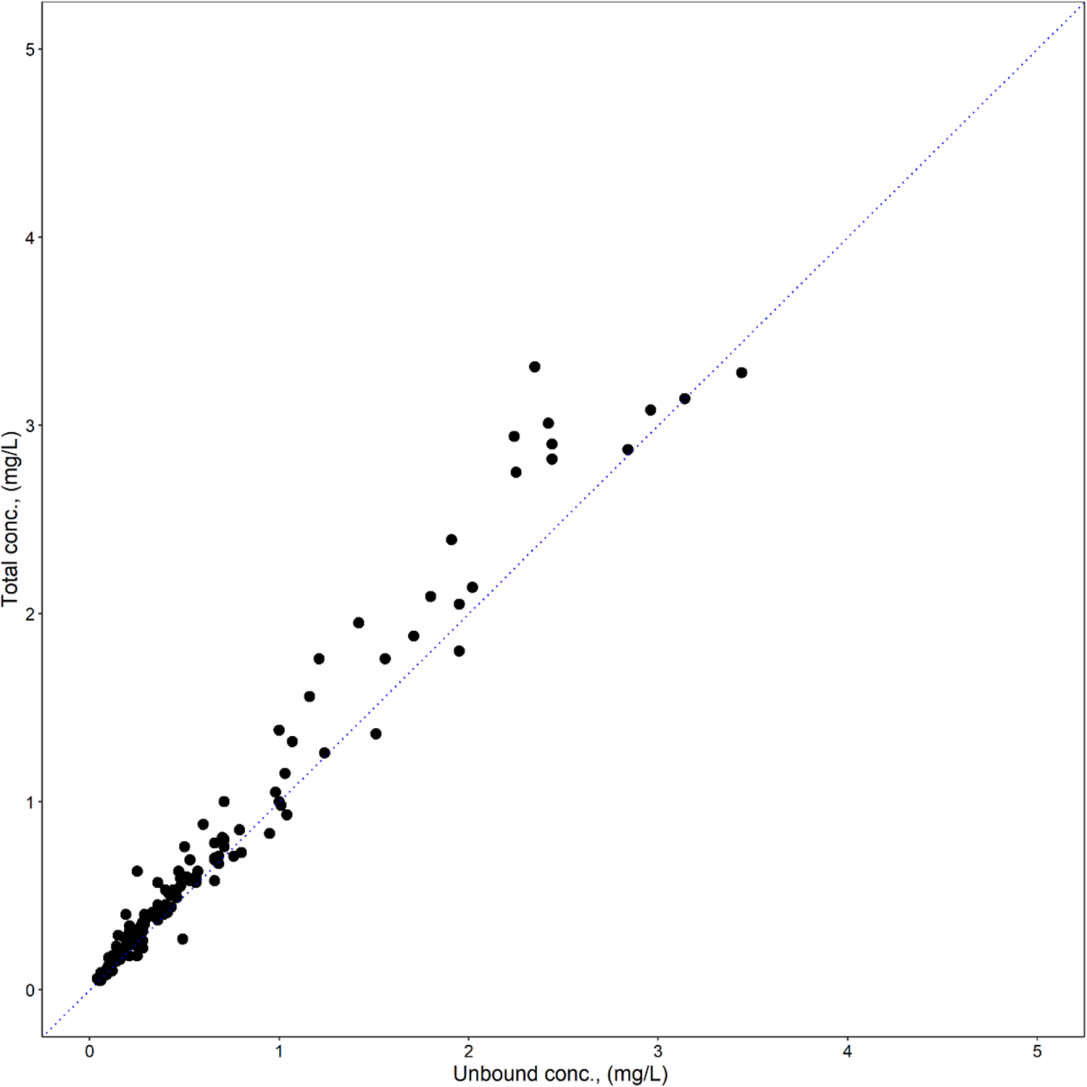
Scatterplot of unbound versus total F-Ara-A concentrations from 22 patients

Since a plot of unbound versus total F-Ara-A concentrations (*C*_unbound_, *C*_total_, respectively) demonstrated a linear association (Fig. 1), a linear protein binding model was implemented for F-Ara-A. *C*_unbound_ and *C*_total_ were modelled simultaneously with *C*_unbound_ as the central compartment. *C*_total_ was predicted from *C*_unbound_ and fraction unbound (*fu*) (Eq. 1):

1 $${C}_{\mathrm{total}}={C}_{\mathrm{unbound}}/fu.$$

The basic structural model was selected by comparing the performance of the two- and three-compartment pharmacokinetic models, assuming first-order kinetics. A one-compartment model was not tested since visual inspection of the concentration versus time plots indicated multi-phase elimination profiles. Inter-individual variability (IIV) was assumed to follow a log-normal distribution. Inter-occasion variability (IOV) was implemented similarly, with each dose defined as a separate occasion. Residual error was evaluated as either proportional, additive or a combination of the proportional and additive error models. Potential predictors of variability in the pharmacokinetic parameters were screened as covariates for inclusion in the model. Continuous covariates were evaluated using a linear or power function described in Eq. 2:

2 $${P}_{\mathrm{i}}={P}_{\mathrm{pop}} {\left({\mathrm{Cov}}_{\mathrm{i}}/{\mathrm{Cov}}_{\mathrm{typical}}\right)}^{\mathrm{k}},$$

where Cov_i_ is the covariate value for the *i*th individual, Cov_typical_ is the typical or median value in the population and *k* is the exponent which was fixed at 1 for a linear function or estimated for a power function. Binary categorical covariates were evaluated using Eq. 3:

3 $${P}_{\mathrm{i}}={P}_{\mathrm{pop}}\left(1+{P}_{\mathrm{cov}}\right),$$

where *P*_cov_ is the estimated proportional factor with which the parameter changes for a specific covariate.

Renal function was incorporated into the model by differentiating total unbound clearance (CLu) into non-renal and renal components as previously described [12]. Body weight was applied to total CLu as previously described [12]. Alternative descriptors of body size (FFM, BSA, BMI, height) were evaluated as a replacement for weight. Height, BSA and BMI were scaled linearly. Allometric scaling factors of 0.75 for unbound CLu parameters and 1.0 for the volume of distribution (Vu) parameters were applied to the weight and FFM covariates, with the weight covariate centred using a reference body weight of 70 kg. The impact of patient maturation (FMAT) was separately evaluated on renal and non-renal clearance using a standard maturational model [30] that incorporated PMA, a sigmoid function (HILL) and the PMA representing 50% renal maturation (PMA_50_):

4 $$FMAT=\frac{ {PMA}^{\mathrm{HILL}}}{{PMA}{_{50}}^{\mathrm{HILL}}+{PMA}^{\mathrm{HILL}}}$$

The Hill exponent was restricted to be less than 5 in order to obtain a plausible maturation curve.

Stepwise forward selection and backward elimination methods using the likelihood-ratio test were employed to determine covariate significance, with covariates selected for inclusion if they decreased the model objective function value (OBV) by more than 3.84 units (*p* < 0.05) during forward selection and 6.63 units (*p* < 0.01) during backward elimination. The most appropriate covariate model was selected based on (1) a low value for the objective function (OBV), (2) low estimates for sigma, (3) low relative standard error estimates for all pharmacokinetic parameters, (4) low estimates of IIV and IOV, (5) low shrinkage of below 30% [31] for the random and residual error components and (6) good model performance as assessed using goodness-of-fit plots, a visual predictive check (*n* = 1000) and non-parametric bootstrapping (*n* = 1000).

### Derived pharmacokinetic parameters

A number of pharmacokinetic parameters for F-Ara-A were derived from the post hoc Bayesian estimates of the primary pharmacokinetic parameters to allow comparison across age groups and GFR categories. CLu and V1u were normalised to WT (70 kg) by dividing by WT and multiplying by 70. Unbound AUC_0-∞_ (AUCu_0-∞_) was determined by dividing the dose (mg) by the individual posterior Bayesian estimates of F-Ara-A CLu. Total AUC_0-∞_ (AUCt_0-∞_) was calculated as AUCu_0-∞_ divided by *fu*. Median and interquartile range for AUCu_0-∞_ and AUCt_0-∞_ were then calculated for mg/m^2^ doses ranging from 10 to 50 mg/m^2^ to provide an indication of the expected range of exposures using current oncology protocols. Percent unbound F-Ara-A was calculated as *fu* multiplied by 100, and percent protein bound F-Ara-A was calculated as (1-*fu*) multiplied by 100.

In order to compare pharmacokinetic results from previously published studies, clearance and volume of distribution parameters were converted to weight- normalised estimates (70 kg) to allow comparison using common units. In addition, total and unbound AUC_0-∞_ were normalised to mg fludarabine dose and allometric size using Eq. 5 and data on mg/m^2^ fludarabine dose and the median values of BSA and WT in the populations studied. For those studies in which median BSA and/or WT were not reported, values of 1.73m^2^ and 70 kg, respectively, were used in the calculations. Linear pharmacokinetics for fludarabine was demonstrated in early studies [32, 33].

5 $${\mathrm{Normalised\, AUC}}_{0-\infty }=\frac{{\mathrm{AUC}}_{0-\infty }}{\mathrm{fludarabine\, dose }\;\left(\text{mg}\right)}\times {\left(\frac{70}{\mathrm{WT}\left(\mathrm{kg}\right)}\right)}^{0.75}.$$

### Impact of age on derived pharmacokinetic parameters for total and unbound F-Ara-A

Patients were sub-divided into groups according to age (< 1 year (*n* = 29), 1–7 years (*n* = 69), 7–12 years (*n* = 38), 12–20 years (*n* = 48), > 20 years (*n* = 27)). GFR (mL/min/1.73 m^2^), F-Ara-A *fu*, CL(L/h/70 kg), V1 (L/70 kg) and normalised AUCu_0-∞_ were then compared using the Kruskal–Wallis H test with post hoc pairwise comparison using the Holm-adjusted Dunns test. Violin plots with super-imposed boxplots with statistical comparisons were implemented in RStudio using the ggstatsplot package [28].

### Evaluation of F-Ara-A exposure and dosing in renal impairment

The Kidney Disease Improving Global Outcomes (KDIGO) 2012 Clinical Practice Guideline for the Evaluation and Management of Chronic Kidney Disease (CKD) [34] categories were applied to the F-Ara-A AUCu_0-∞_ and CLu values, as recommended in the International Consensus Guidelines on Anticancer Drug Dosing in Kidney Dysfunction (ADDIKD) [18]. These categories were GFR ≥ 90 mL/min/1.73 m^2^ (G1, normal or high GFR), GFR 60–89 mL/min/1.73 m^2^ (G2, mildly decreased GFR), GFR 45–59 mL/min/1.73 m^2^ (G3A, mildly-moderately decreased GFR) and GFR 30–44 mL/min/1.73 m^2^ (G3B, moderately-severely decreased GFR). Percent difference of median CLu (L/h/70 kg) for the G2, G3A and G3B categories when compared with the G1 category was then calculated using Eq. 6:

6 $$\mathrm{\%\, difference\, in\, CLu }=\frac{\mathrm{CLu\, }\left(\mathrm{G1\, category}\right)-\mathrm{CLu\,}(\mathrm{G2,G3\, categories})}{\mathrm{Clu\, }\left(\mathrm{G1\, category}\right)}\times 100$$

In nine individuals with GFR < 60 mL/min/1.73 m^2^, the mg fludarabine dose required to target the median AUCu_0-∞_ observed for the relevant mg/m^2^ dose in the treatment protocol was calculated using Eq. 7 and compared to the mg fludarabine dose calculated using BSA-based dosing. A percent difference in dose within 20–25% reflects the attainment of the target AUCu_0-∞_ within acceptable limits, the 80–125% interval [35, 36].

7 $$\mathrm{Dose }\left(\mathrm{mg}\right)\mathrm{to\,target\ AUCu}_{\mathrm{o}-\infty }=\mathrm{administered }\left(\mathrm{mg}\right)\mathrm{ dose}\times \frac{\mathrm{target\, AUCu}_{\mathrm{o}-\infty } }{\mathrm{observed\, AUCu}_{\mathrm{o}-\infty }}$$

## Results

### Patients

The clinical data for the patients in this study is shown in Table 1. A total of 211 patients were recruited between 2005 and 2021 (64% male, 36% female). Patient age ranged from 0.11 to 63.4 years (median 7.62 years) and 14% of the population were less than 1 year of age. The population included patients that varied widely in patient weight (range, 2.9 to 124.3; median 24.4 kg) and GFR (range, 30 to 300; median 110.7 mL/min/1.73 m^2^). Most patients were being treated for haematological malignancies (58%); the remainder had inborn errors of immunity (22%) or other genetic diseases (20%).

### F-Ara-A concentration data

Unbound F-Ara-A concentration data included 1403 observations from 211 patients: 777 observations (55%) were timed from 0 to 5 h post infusion, 538 observations (38%) were timed from between 5 and 12 h and 88 observations (6%) were timed at greater than 12 h after the infusion. The majority of unbound concentration data (n = 1062, 76%) was collected on the first day of treatment. Total F-Ara-A concentration data included 2480 observations from 211 patients; 1328 observations (54%) were timed from 0 to 5 h after the infusion, 1033 observations (41%) were timed from between 5 and 12 h, and 119 observations (5%) were timed at greater than 12 h after the infusion. For each dosing occasion, 1144 observations were taken during day 1 (46%), 492 on day 2 (20%), 437 on day 3 (18%), 364 on day 4 (15%) and 42 on day 5 (1%).

### Structural and statistical model for F-Ara-A (Base model)

A three-compartment linear protein binding model, parameterized in terms of unbound volume of distribution into central V1u and peripheral (V2u, V3u) compartments and clearance from the central compartment (CLu) and between V1u and V2u (Q2u) and between V2u and V3u (Q3u), produced the lowest OBV and described the data better than a two-compartment linear binding model (the decrease in OBV (dOBV) was 361). Allometric weight was included as a covariate on all (intercompartmental) clearances and volumes of distribution during model development. As previously observed [12], the introduction of IIV and IOV to all of the CLu, Q2u and Q3u parameters and the V1u, V2u and V3u parameters combined improved model stability and resolved the issue of a high degree of correlation of random effects. A proportional error model best described the residual error. The population pharmacokinetic parameter estimates for the base model are shown in Table 2.

**Table 2:** Population pharmacokinetic parameter estimates for the three-compartment model for F-Ara-A based on unbound plasma concentrations

	**Base model**	**Covariate Model**	
**Pharmacokinetic parameter**	**Population mean (%RSE) [shrinkage%]**	**Population mean (%RSE) [shrinkage%]**	**Bootstrap estimate, mean (95% CI)**
Structural model for F-Ara-A clearance: $CLu=\left(CLu renal*FMATR+CLu non-renal\right)*{\left[\frac{WT\left(kg\right)}{70}\right]}^{0.75}$, where $$\begin{array}{cc}CLu renal=slope*\frac{GFR}{90}\left(\frac{ml}{min}\right)*\frac{70}{WT\left(kg\right)}& FMATR=\frac{ {PMA}^{HILL}}{{PMA50}^{HILL}+{PMA}^{HILL}}\end{array}$$			
CLu(L/h/70 kg)	3.42 (3%)		
CLuNR (L/h/70 kg)		1.81 (9%)	1.77 (1.31 – 2.16)
Slope		1.02 (9%)	1.06 (0.83 – 1.42)
FMATR (weeks)			
Hill exponent		2.97 (4%)	2.83 (0.99 – 4.66)
PMA_50 _(weeks)		69.1 (8%)	70.5 (47.2 – 92.1)
V1u (L/70 kg)	9.65 (8%)	9.29 (6%)	9.19 (6.95 – 10.32)
Q2 u(L/h/70 kg)	6.54 (24%)	7.48 (15%)	7.83 (5.61 – 14.07)
V2u (L/70 kg)	8.17 (9%)	8.53 (7%)	8.54 (7.13 – 10.45)
Q3u (L/h/70 kg)	1.47 (16%)	1.51 (7%)	1.52 (1.32 – 1.78)
V3u (L/70 kg)	16.4 (10%)	18.6 (11%)	18.8 (15.4 – 23.8)
*fu*	0.877 (1%)	0.878 (1%)	0.878 (0.858 – 0.899)
IIV on CLu, Q2u, Q3u (%)	37.5 (9%) [6%]	33.9 (11%) [7%]	33.7 (27.0 – 40.8)
IIV on V1u, V2u, V3u (%)	40.1 (9%) [10%]	40.6 (10%) [11%]	40.2 (32.5 – 47.7)
IIV on FU (%)	13.7 (10%) [17%]	13.7 (10%) [17%]	13.7 (10.6 – 16.5)
IOV on CLu,Q2u,Q3u (%)	13.5 (12%) [38%]	13.2 (11%) [38%]	13.2 (10.4 – 16.2)
IOV on V1u, V2u, V3u (%)	17 (19%) [45%]	17.5 (17%) [45%]	17.2 (9.1 – 23.1)
σ_PROP_ (Cunbound)	18.5 (8%) [9%]	18.4 (8%) [9%]	18.3 (15.6 – 20.9)
σ_PROP_ (Ctotal)	17.2 (6%) [10%]	17.1 (6%) [10%]	17.1 (15.3 – 19.1)
OBV	-16,011.773	-16,108.785	-16,180.97

**Abbreviations:** *Unbound* u, *CLu* clearance, *V1u* Volume of distribution into the central compartment, *Q2u and Q3u *Intercompartmental clearance parameters, *V2u and V3u* Volume of distribution into the peripheral compartments, CL and V parameters were centred at 70 kg with allometric exponents of 0.75 and 1.0, respectively. *fu* fraction unbound, *CLu renal* renal clearance, *CLu non-renal* non renal clearance, *GFR* glomerular filtration rate, *FMATR* maturation factor for renal clearance, *PMA* post-menstrual age (weeks), *PMA*_*50*_ postmenstrual age at 50% maturity. *RSE* relative standard error (mean %), *IIV* intersubject variability, *IOV* inter-occasion variability and residual error (σ) components are presented as %coefficient of variation (%CV). Bootstrap mean and 95% confidence interval (95% CI) estimates are based on 1000 bootstrap simulations. OBV = objective function value

### Covariate model for F-Ara-A

Inclusion of GFR significantly improved the model fit and reduced the OBV (dOBV = 101.5). Renal and non-renal clearance were differentiated from each other as shown in Eq. 8, with renal clearance estimated as GFR multiplied by a slope estimate (unitless) that represents the fraction of GFR that accounts for renal F-Ara-A clearance (Eq. 9). Inclusion of a sigmoidal patient maturation factor on renal clearance (FMATR) further reduced the OBV (dOBV = 27.85). No other significant covariate relationships could be identified.

8 $$C\mathrm{Lu}=\left(\mathrm{CLu renal}*\mathrm{FMATR}+\mathrm{CLu non}-\mathrm{renal}\right)*{\left[\frac{\mathrm{WT}\left(\mathrm{kg}\right)}{70}\right]}^{0.75}$$

where

9 $$\mathrm{CLu renal}=\mathrm{slope}*\frac{\mathrm{GFR}}{90}\left(\frac{\mathrm{ml}}{\mathrm{min}}\right)*\frac{70}{\mathrm{WT}\left(\mathrm{kg}\right)}$$

The population pharmacokinetic parameter estimates from the final covariate three compartment linear protein binding model for F-Ara-A are shown in Table 2. The NONMEM control file for the final model is provided in the Supplementary Material.

## Model evaluation

**Fig. 2:**
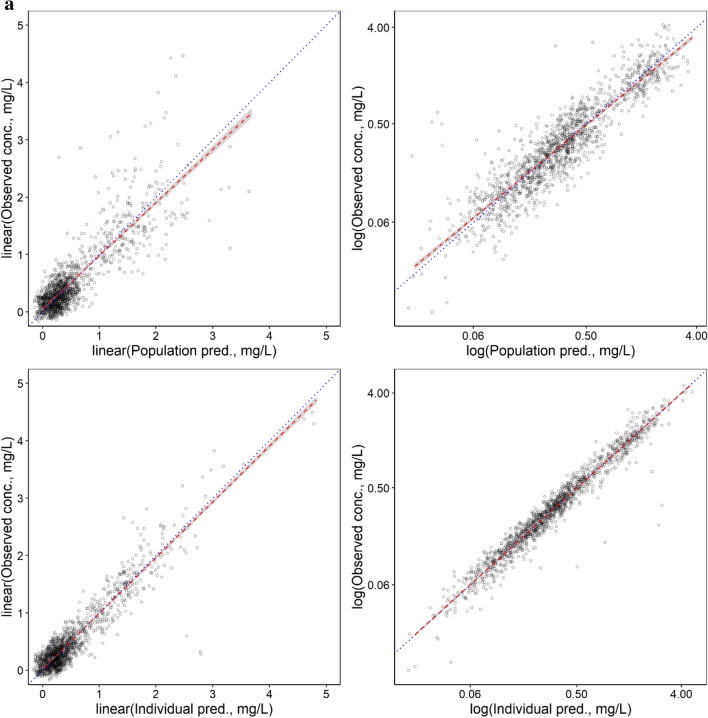

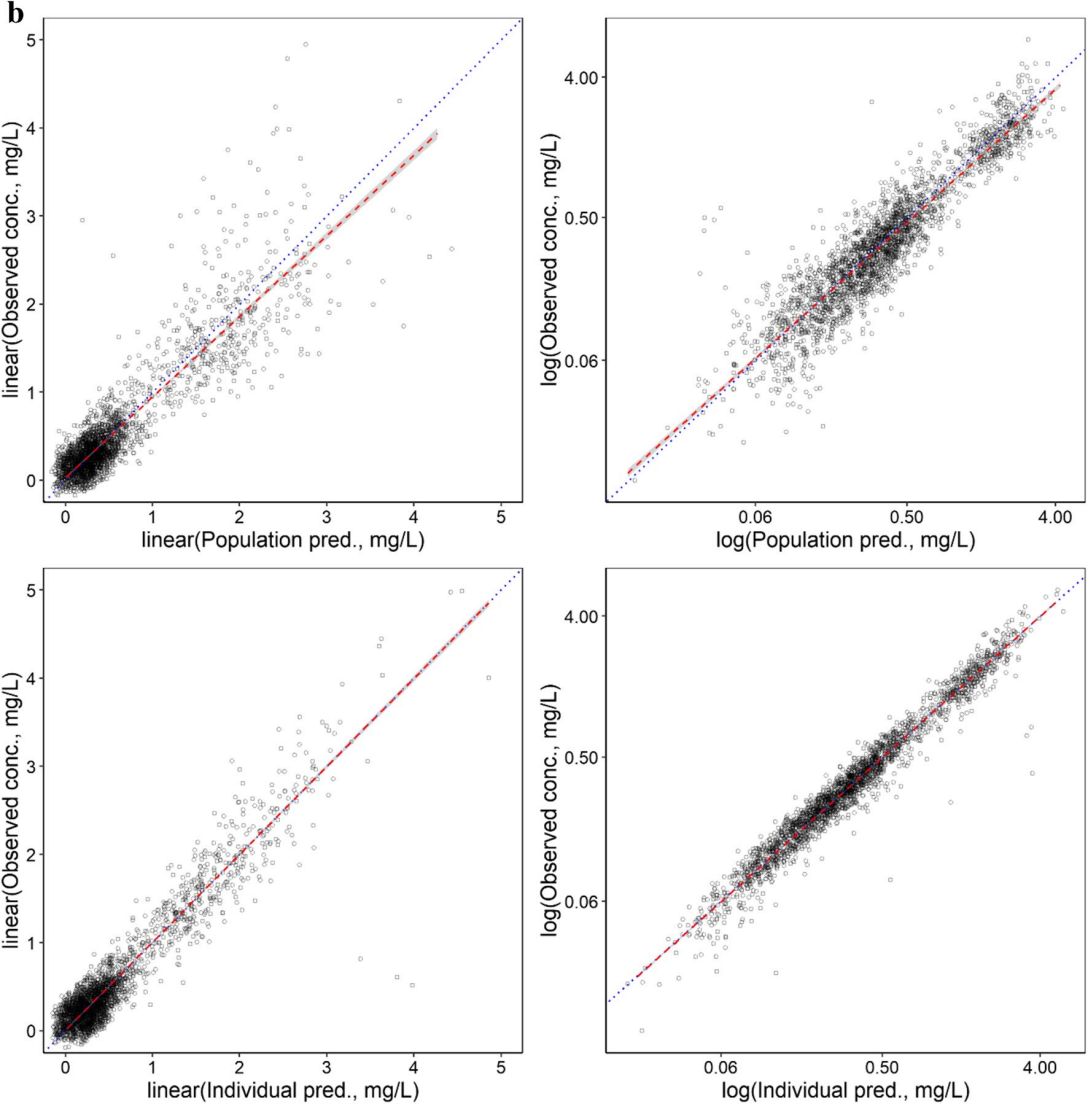
Population and Individual -predicted versus observed concentrations for (**a**) unbound F-Ara-A s and (**b**) total F-Ara-A using the three compartment linear protein binding model that included covariates

**Fig. 3:**
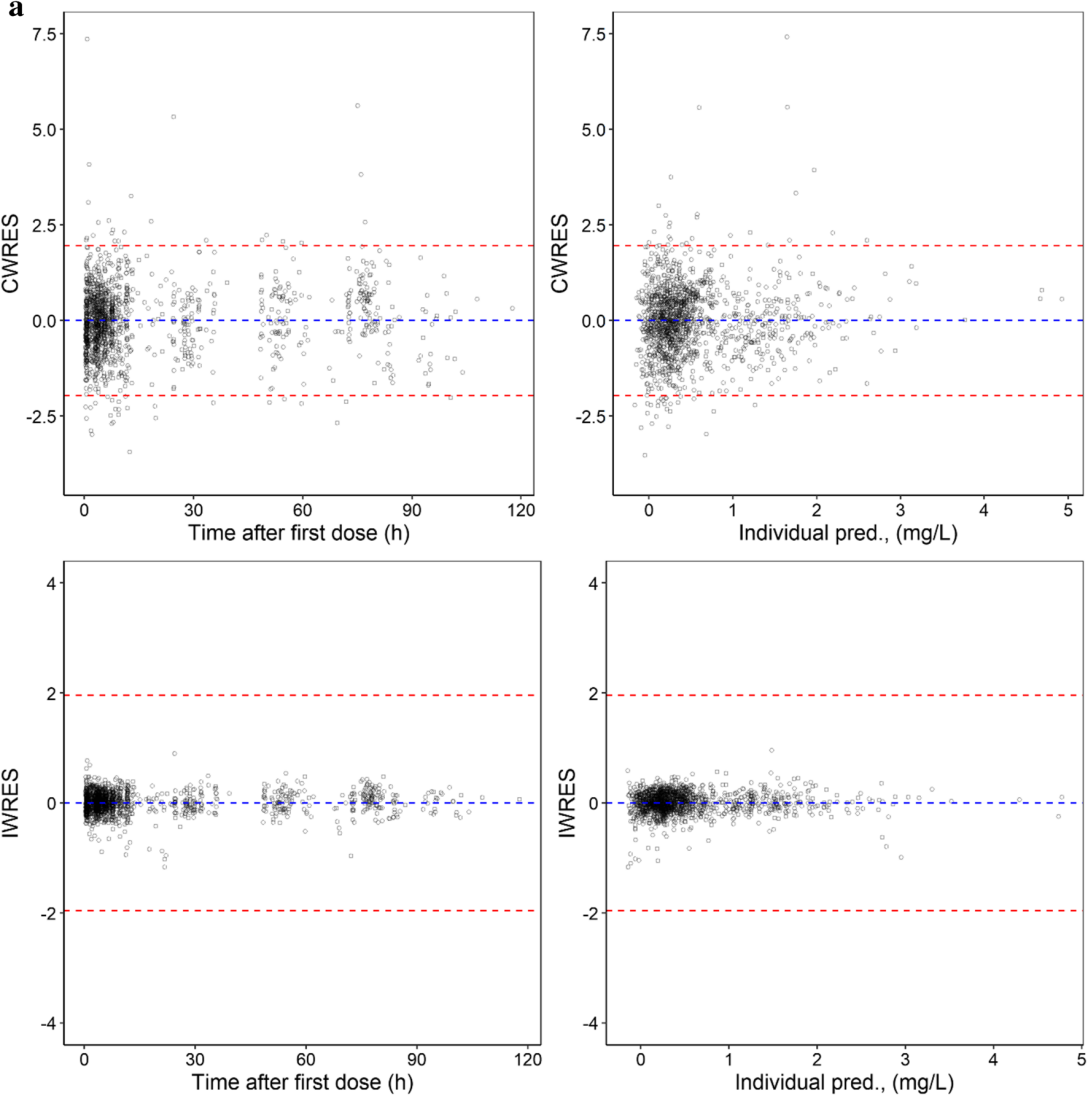

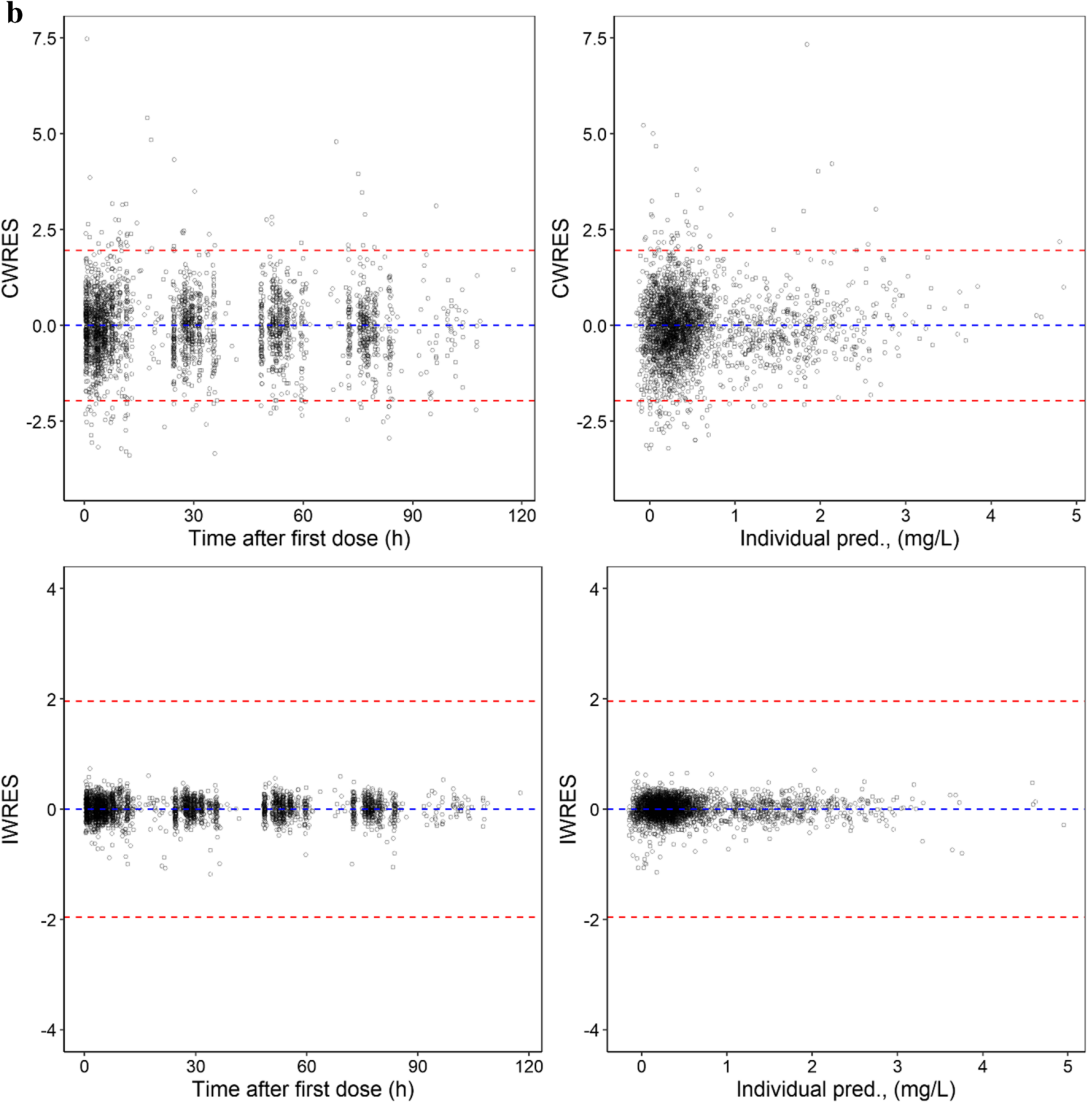
Residual plots for (**a**) unbound F-Ara-A s and (**b**) total F-Ara-A using the three compartment linear protein binding model that included covariates

**Fig. 4:**
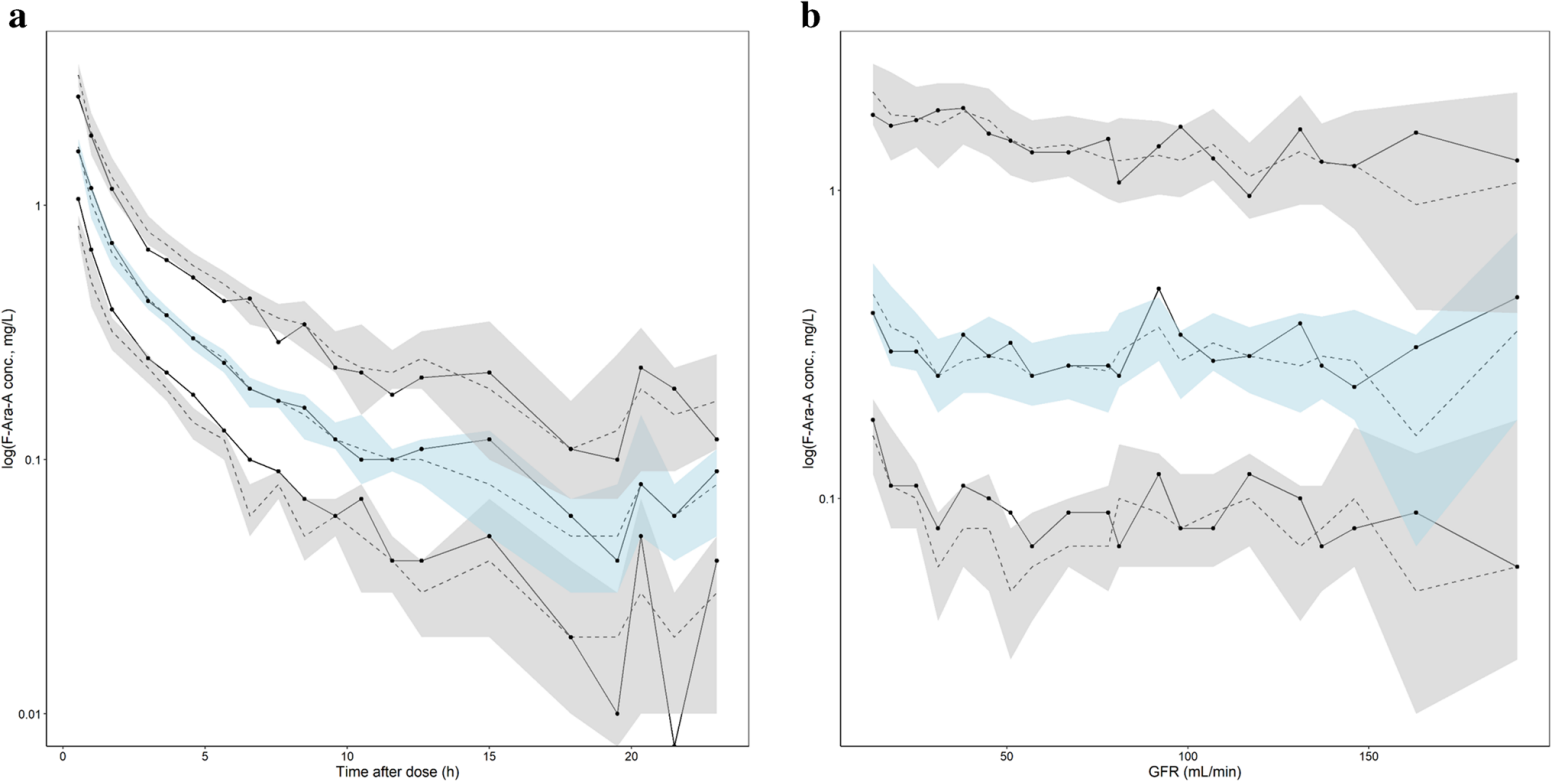
Standard visual predictive check for the three-compartment linear protein binding pharmacokinetic model for F-Ara-A with **(a)** time after dose and **(b)** GFR as independent variables. The unbroken lines show median observed F-Ara-A concentrations at the 10^th^, 50^th^ and 90^th^ percentiles, whilst the dashed line shows the median simulated concentrations with 95% confidence interval (shaded areas)

As shown in Table 2, low RSE values were observed for the unbound F-Ara-A pharmacokinetic parameter estimates and IIV values, and mean bootstrapping results were comparatively similar to the final estimates and within the 95% confidence interval indicating a precise and suitably parameterised model. The goodness of fit plots demonstrated accurate population and individual predictions for both unbound and total F-Ara-A concentrations (Fig. 2a and b, respectively). The plots of conditional weighted residuals (CWRES) and individual weighted residuals (IWRES) versus time and the individual predictions were normally distributed and centred at 0 with variance ω^2^ for both unbound and total F-Ara-A (Fig. 3a and b, respectively). The final model was also determined to be sufficiently robust as indicated by the simulation-based visual predictive checks with time after dose and GFR as the independent variables (Fig. 4a and b, respectively).

## Exposure distribution – comparison with previous studies

In Table 3, our results for total and unbound F-Ara-A pharmacokinetic parameters are compared with those obtained in previous studies. In adults, our values compared most closely with those obtained in the phase 1 clinical trial conducted by Malspeis et al. [15]: the median values values for normalised AUCu_0-∞_ were 0.10 versus 0.11 mg/L.h, respectively, and the median values for CLu were 6.5 and 6.9 L/h/70 kg, respectively. Similar to us, this study measured unbound F-Ara-A concentrations and employed a three-compartment model. Previous adult studies that utilised two compartment pharmacokinetic models [14, 16] tended to have higher clearance and lower normalised AUCt_0-∞_ results compared with our results. Our paediatric data was most consistent with that of the study of Chung et al. [9] with median CL results of 12.2 and 12.6 L/h/70 kg, respectively, with wide variability observed in both studies. In datasets that combined results from adults and children, our median CL results were similar to those obtained using the three compartment model developed by Langenhorst et al. [12] (11.5 versus 12.6 L/h/70 kg, respectively), however their normalised AUCt_0-∞_ results tended to be lower and there were discrepancies with the population mean estimates of the pharmacokinetic parameters. Mohanan et al. [13] also observed a wide variability in CL (4.2—60.8 L/h/70 kg), with differences observed between patients with aplastic anaemia (median CL = 17.8 L/h/70 kg) and Fanconi anaemia (median CL = 7.4 L/h/70 kg).

**Table 3:** Total and unbound F-Ara-A pharmacokinetic parameters – comparison with other studies in adults and children

**Study**	**Age range (years)**	**GFR range** **(ml/min/1.73m**^**2**^**)**	**Daily fludarabine dose (mg / mg/m**^**2**^**)**	**N**	Dose and allometric size normalised **AUC**_**(0-∞)**_** (mg/L.h)**	**CL / CLu** **(L/h/70 kg)**	**V1, V2, V3 /** **V1u, V2u, V3u** **(L/70 kg)**
**Adults**							
This study#	36 -63	69 – 127	75 (30 – 90) mg 39 (21– 52) mg/m^2^	27	AUCt_(0-∞)_: 0.12 (0.06 – 0.36) AUCu _(0-∞):_ 0.10 (0.05 – 0.30)	CL: 6.0 (4.2 – 11.3) CLu: 6.5 (4.5–12.4)	V1u: 20 (9 – 40) V2u: 19 (9–37) V3u:41 (19–80)
Malspeis et al. [15] # a	Not provided	Normal (n = 22) Impaired renal function (n = 7)	208 (138 – 450) mg 120 (80–260) mg/m^2^	**30**	AUCu _(0-∞)_: 0.11 (0.07 – 0.27)	CLu: 6.9 (3.8 – 16.6)	V1u: 12.1 (6.6 – 28.6) V2u: 21.0 (5.6–35.4) V3u:40.8 (19.5 –81.3)
Bonin et al. [7]$	33 – 66	Normal	58 mg (median) 30 mg/m^2^	16	AUCt_(0-∞)_: 0.07 (0.03 – 0.13)	CL: 7.6 (4.0 – 15.0)	
Sanghavi et al. [14]*	20—69	45 – 153 ml/min	78 mg (median): 40 mg/m^2^	87	AUCt_(0-∞)_: 0.04 (0.02 – 0.06)	CL: 13.7 (8.8 – 23.5) **Population estimates:** CLNR:7.04 ± 14.1% CLslope:3.9 ± 25.2% Q2: 9.52 ± 6.2%	**Population estimates:** V1: 65.9 ± 2.9% V2: 67.2 ± 6.7%
Long-Boyle et al. [16]$ a	20 – 69	50–153 ml/min	69 mg (median) 40 mg/m^2^	78	AUCt_(0-∞)_: 0.05 (0.02 – 0.11)	CL:12.0 (5.1 – 29.3)	
**Children**							
This study#	0.1 – 19.9	30—300	24 (5- 100) mg 30 (10–52) mg/m^2^	184	AUCt_(0-∞)_: 0.56 (0.04 – 23.0) AUCu _(0-∞)_: 0.49 (0.04 – 19.9)	CL: 12.2 (1.9 – 31.2) CLu: 13.8 (2.0–37.1)	V1u: 27 (3 – 90) V2u: 25 (3–83) V3u: 54 (6 –180)
Ivaturi et al. [11]*	0.2 – 17.9	35–150 ml/min/m^2^	40 mg/m^2^, n = 55 12.5–35 mg/m^2^, n = 40 0.9– 1.33 mg/kg, n = 38	140	AUCt_(0-∞)_: 0.55 (0.45 – 0.59) (n = 55, 40 mg/m^2^ dose)	CL = 27.0 (25 – 33), n = 55 **Population estimates** CL = 14.5 ± 3.3% Q2 = 5.7 ± 10.4%	**Population estimates:** V1 = 34.5 ± 6.3% V2 = 62.5 ± 4.8%
Chung et al. [9] *	1.3 – 18	81 – 299	52 mg (median) 40 mg/m^2^	41	AUCt_(0-∞)_: 0.12 (0.08 – 0.27)	CL:12.6 (4.9 – 31.0) **Population estimates:** CL:11.3 ± 4.1% Q2: 10.2 ± 8.1%	**Population estimates:** V1: 40.3 ± 4.1% V2: 47.3 ± 5.2%
**Adults and Children combined**							
This study	0.11 – 63.4	30—300	27 (5 – 100) mg 30 (10 – 50) mg/m^2^	211	AUCt_(0-∞)_: 0.44 (0.04 -23.0) AUCu _(0-∞)_: 0.36 (0.04 – 19.9)	CL: 11.5 (1.9 – 31.2) CLu: 13.1 (2.0–37.1) **Population estimates**: CLu = 3.42 ± 3% CLuNR = 1.81 ± 9% CLRu = 1.02 ± 9% Q2u:7.48 ± 15% Q3u:1.51 ± 7%	V1u: 26 (3 – 90) V2u: 24 (3 – 83) V3u: 53 (6 – 180) **Population estimates:** V1u: 9.29 ± 6% V2u: 8.53 ± 7% V3u: 18.6 ± 11%
Langenhorst et al.[12] # a	0.3 – 74	25 – 140	69 mg (median) 40 mg/m^2^ (n = 197) 10 mg/m^2^ (n = 61)	258	AUCt_(0-∞)_: 0.08 (0.03 -0.21)	CL: 12.6 (4.9 – 31.0) **Population estimates:** CLNR: 3.24 ± 20% CLR = 0.782 ± 11% Q2:8.6 ± 8% Q3:3.8 ± 13%	**Population estimates:** V1: 39 ± 5% V2: 20 ± 11% V3: 50 ± 9%
Mohanan et al. [13] *	3 – 57		45 mg (median) 30 mg/m^2^	53	AUCt_(0-∞)_: AA: 0.08 (0.02 – 0.35) FA: 0.20 (0.14 – 0.35)	**CL:** AA: 17.8 (4.2 – 60.8) FA: 7.4 (4.2 – 10.8) **Population estimates:** CL: 4.55 ± 9.8%	**Population estimates:** V1:25.9 ± 6.4%

Three (#), Two (*) and non ($) compartment modelling were used for pharmacokinetic parameter determination. Data are median (range) except when population mean ± RSE is presented. *N* number of patients in group, *u* unbound, *CL* clearance, *CLNR* non-renal clearance, *CLR* renal clearance, *V1* Volume of distribution into the central compartment, *Q2 and Q3* Intercompartmental clearance parameters, *V2 and V3* Volume of distribution into the peripheral compartments. AUCt_(0-∞)_ and AUCu _(0-∞)_ are total and unbound F-Ara-A AUC, normalised to mg fludarabine dose and allometric size. *AA* aplastic anaemia, *FA* Fanconi anaemia, ^a^ Fludarabine dose (mg) and parameters normalised to allometric size were estimated using a median BSA of 1.73 m^2^ and /or median weight of 70 kg due to lack of information in the original manuscript

## Protein binding, *fu* and exposure determinations in the population

The median (range) value of *fu* generated by the model for the entire patient cohort was 0.89 (0.57 – 1.09), with percent protein-unbound F-Ara-A calculated to be 89 (57 to 109)% and percent F-Ara-A protein-bound calculated to be 11 (-9 to 43)%. 16 patients (8%) had *fu* estimates that were above the theoretical maximum of 1.0. These model-generated estimates for *fu* were consistent with those obtained by simply dividing the unbound concentration by the total concentration for each sample tested and obtaining a mean *fu* for each patient: the median and range were 0.90 (0.47 – 1.22). The observed median (interquartile range) for AUCu_0-∞_ and AUCt_0-∞_ for fludarabine doses ranging from 10 to 50 mg/m^2^ are shown in Table 4.

**Table 4:** Observed F-Ara-A exposure for fludarabine doses ranging from 10 to 50 mg/m^2^

mg/m^2^ dose	n	AUCu_0-∞_	AUCt_0-∞_
10	6	2.3 (1.7 – 5.0)	2.8 (2.3 – 5.8)
25	32	3.9 (2.8 – 5.6)	4.6 (3.3 – 6.6)
30	109	4.6 (3.6 – 5.6)	5.0 (4.2 – 6.4)
40	56	6.6 (5.5 – 8.6)	7.5 (6.2 – 9.4)
50	8	9.4 (8.6 – 14.7)	11.1 (9.5 – 17.5)

Data are median (interquartile range), n = number of patients per dose group

AUCu_0-∞_ and AUCt_0-∞_ are unbound and total F-Ara-A AUC_0-∞_, respectively

### Age-related differences in F-Ara-A* fu* and pharmacokinetic parameters

**Fig. 5:**
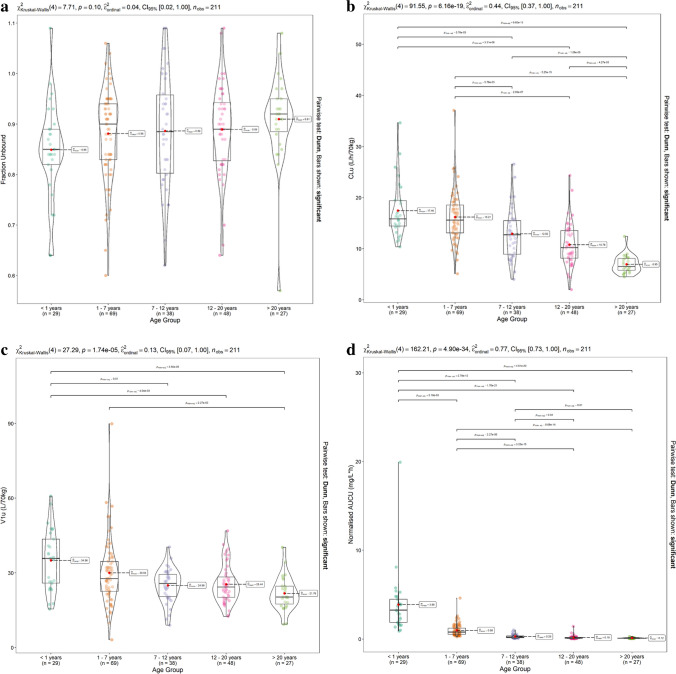

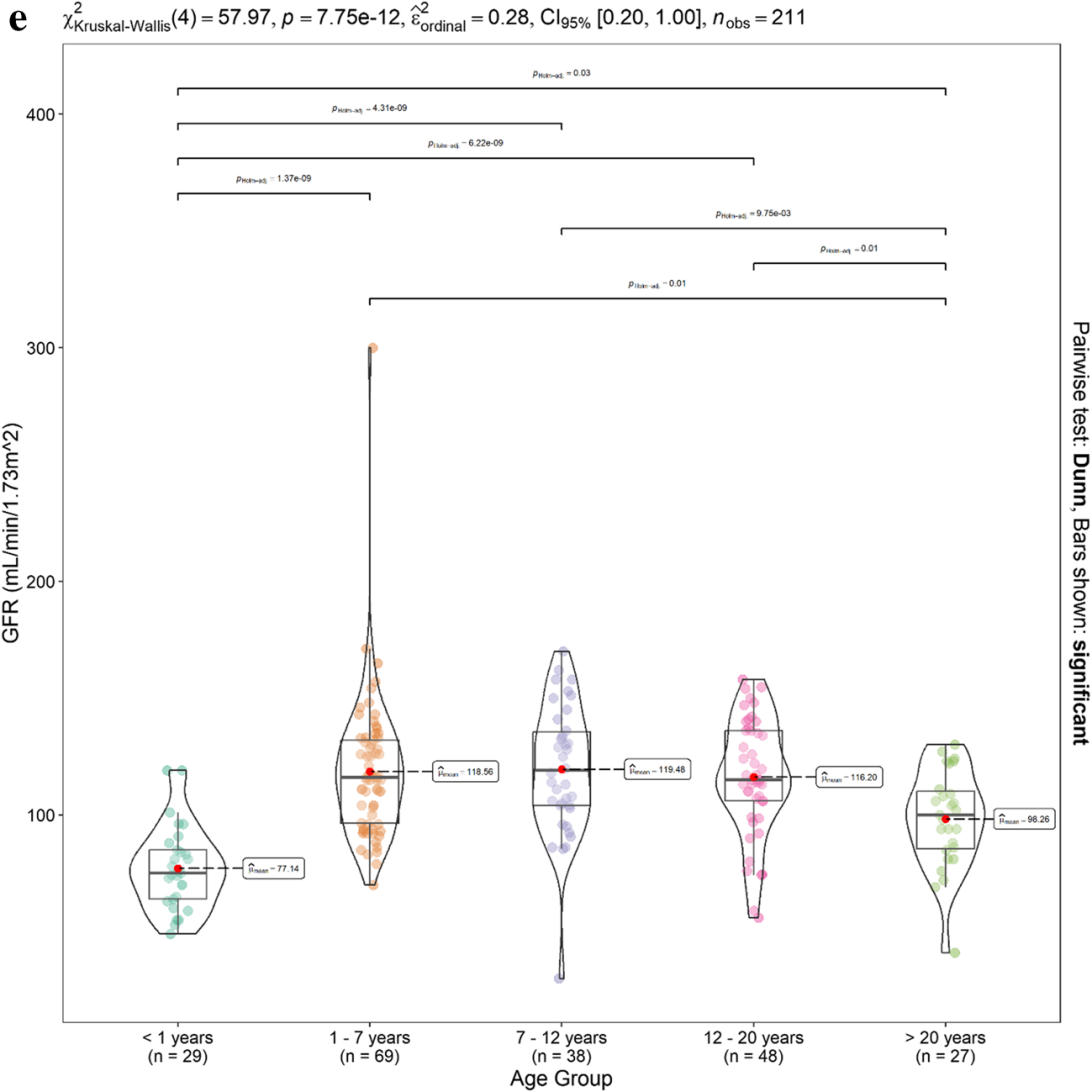
Association of age-group on F-Ara-A (**a**) *fu* (**b**) CLu (L/h/70 kg), (**c**)V1u (L/70 kg) and (d) AUCu_0-∞_ normalised to mg fludarabine dose and allometric size and (**e**) GFR (ml/min/1.73 m.^2^)

The Kruskal–Wallis H test showed that F-Ara-A *fu* did not differ significantly between age groups (Fig. 5a). F-Ara-A CLu (L/h/70 kg) and V1u (L/70 kg) were significantly higher in the younger age groups (p < 0.01, Fig. 5b and c respectively). Normalised AUCu_0-∞_ differed significantly between the majority of age groups (p < 0.01, Fig. 5d). Both adults (> 20y) and infants (< 1 y) were characterised by significantly reduced renal function, when compared with the remaining age groups, p < 0.01 (Fig. 5e).

### Evaluation of F-*Ara*-A clearance, exposure and dosing in renal impairment

Table 5 shows the impact of renal impairment on unbound F-Ara-A clearance and exposure. When compared with patients with G1 KDIGO category, median CLu (L/h/70 kg) was 11% and 54% lower for patients in the G3A and G3B categories, respectively, whilst there was minimal difference (5% increase) for patients in the G2 category. The impact of reduced GFR on the 9 individuals who had GFR values < 60 ml/min/1.73m^2^ is shown in Table 6. The two patients (aged 11.4 and 43.6 y) with a GFR in the 30 – 44 ml/min/1.73m^2^ range (G3B category), who received reduced doses of 52% and 47%, respectively, achieved AUCu_0-∞_ values within ± 14% of the target value. Among the 7 patients with GFR values between 45 and 59 ml/min/1.73 m^2^ (G3A category), three patients (IDs 47, 129, and 161) would have required dose reductions of 43% to 54% to reach the target AUCu _0–∞_. Three patients (IDs 7, 6, and 32), aged between 0.21 and 13.7 years, would have achieved AUCu _0–∞_ values within ± 14% of the target without dose adjustments, while one patient aged 0.82 years (ID 118) would have needed a dose increase to meet the target value.

**Table 5:** Impact of renal impairment on unbound F-Ara-A clearance and exposure

	**Total group** **(n = 211)**	**KDIGO category for GFR (ml/min/1.73m**^**2**^**)**			
		**G1**	**G2**	**G3A**	**G3B**
		** > 90 (n = 162)**	**60–89 (n = 40)**	**45–59 (n = 7)**	**30–44 (n = 2)**
Age	8.1 (0.1 – 63.4)	8.8 (0.27 – 63.4)	1.4 (0.2 – 62.7)	0.5 (0.1 -17.4)	27.5 (11.4 – 43.6)
WT	24.4 (2.9 -124.3)	29.4 (6.5 – 124.3)	11.2 (5.0 -101.9)	5.2 (2.9 – 65.5)	38.1 (28.1 – 48)
Dose (mg)	27 (5 – 100)	30 (5 – 100)	17.8 (6.2 – 90)	8.3 (5.0 – 50.0)	21 (12 – 30)
Dose (mg/m^2^)	30 (9.7 – 51.5)	30 (9.7–51.5)	31.5 (19.0 – 51.3)	28.2 (21.2 – 48.4)	16.5 (11.9 – 21.1)
CLu (L/h)	4.50 (0.43 – 18.62)	5.04 (0.7 – 18.6)	2.47 (0.96 – 11.73)	1.40 (0.43 – 6.88)	3.1 (2.79 – 3.42)
CLu (L/h/70 kg) %difference	13.1 (2.0 – 37.1)	13.1 (2.0 – 37.1)	13.8 (4.0 – 28.6) 5%	11.67 (4.5 – 19.4) -11%	5.97 (4.99 – 6.95) -54%
AUCu_0-∞_ (mg/L.h)	5.1 (1.6 – 17.3)	4.73 (1.6 – 17.2)	6.10 (1.9 – 17.3)	5.80 (4.64 – 9.15)	5.1 (3.36 – 6.85)

*KDIGO* kidney disease improving global outcomes, *GFR* glomerular filtration rate, *WT* weight, *CLu* unbound F-Ara-a clearance, *AUCu*_0-∞_ unbound F-Ara-A AUC_0-∞_

Data are median (range), %difference = % diff of median value when compared to median value in the G1 (> 90 ml/min/1.73 m^2^) group

**Table 6:** Evaluation of Fludarabine dosing in 9 patients with GFR < 60 ml/min/1.73m^2^ (G3A and G3B KDIGO categories)

ID	GFR (ml/min /1.73m^2^)	Age (ys)	WT (kg)	Protocol -based Flu dose mg (mg/m^2^)	Administered Flu mg dose (%diff from protocol)	Target AUCu_0-∞_ (mg/L.h)	Observed AUCu_0-∞_ (%diff from Target)	Flu mg dose to achieve target (%diff from protocol)
174	30.00	11.41	28.1	25.3 (25)	12 (-52%)	3.9	3.36 (-13.9%)	14 (-44.8%)
1012	41.00	43.59	48	56.8 (40)	30 (-47%)	6.6	6.83 (3.5%)	29 (-49%)
47#	49.00	.11	2.9	8 (40)	5 (-37.5%)	6.6	8.92 (35.2%)	4 (-54%)
7#	53.00	.21	5.2	11.6 (40)	8.3 (-28.5%)	6.6	4.65 (-29.6%)	12 (1.6%)
129*	55.00	.50	4.5	7.8 (30)	5.6 (-28.2%)	4.6	5.79 (25.9%)	4 (-43%)
6#	55.00	.27	4.9	11.2 (40)	8 (-28.6%)	6.6	5.21 (-21.1%)	10 (-9.5%)
32	56.00	13.70	53.6	47.1 (30)	50 (6.2%)	4.6	5.68 (23.5%)	40 (-14.0%)
161	59.00	17.40	65.5	44.5 (25)	45 (1.2%)	3.9	8.29 (112.6%)	21 (-52%)
118	59.00	.82	7.5	18.5 (50)	18 (-2.7%)	9.4	6.76 (-28.1%)	25 (35%)

*Flu* fludarabine, *GFR* glomerular filtration rate, *WT* weight, %diff = % difference

1.25* and 1.6# mg/kg doses were administered instead of the 30 and 40 mg/m^2^ doses respectively

Target AUCu_0-∞_ corresponds to median observed AUCu_0-∞_ for the relevant protocol-based mg/m^2^ fludarabine dose (refer to Table 4)

## Discussion

This study has, for the first time, described a 3-compartment linear protein binding pharmacokinetic model for F-Ara-A that also allowed estimation of unbound pharmacokinetic parameters estimates and *fu* from the combination of total and unbound concentrations. Given the wide range of *fu* values (0.57–1.09) observed in this population, measurement of unbound exposure will be essential for future pharmacodynamic evaluations aimed at optimising dose. Whilst this model was structured in a similar way to a previously reported three compartment model for F-Ara-A [12], there were discrepancies in the final population pharmacokinetic parameter values, in particular the central and peripheral V parameters. In the previous study [12], in which total F-Ara-A concentrations were analysed, there was sparse data between 0 and 5 h after the infusion end, whilst, in our study, 54% of the drug concentrations were timed within this period. Studies that analysed total F-Ara-A pharmacokinetics using non-compartmental methods [7, 14] or two-compartment models [9, 11], tended to under-estimate F-Ara-A AUC_0-∞_ and over-estimate F-Ara-A clearance when compared with our study. The final pharmacokinetic results reported in our study most closely resembled those obtained in the phase 1 study of Malspeis et al. [15] that also employed a three-compartment model for pharmacokinetic assessment and was characterised by rich unbound F-Ara-A concentration data collection throughout both the distributional and elimination phases. In the product monograph for fludarabine it is noted that discrepancies between the studies regarding the biphasic or triphasic elimination patterns appear to be due to differences in sampling schedules, with sampling duration of 24 to 30 h resulting in a terminal elimination half-life of 8 to 10 h, and longer sampling up to 72 h resulting in values of up to 31 h [33]. Further improvements in the accuracy of population pharmacokinetic models for F-Ara-A might therefore be achieved with additional concentration data in the 12-24 h sampling window and beyond. The visual predictive checks for our model were excellent up until approximately 12 h after dose administration, after which there was increased variability, due in part to the fact that the 12–24 h window was characterised by sparse observed data and F-Ara-A concentration data that was at or below the limit of quantification of our HPLC assay (LOQ:0.1 µg/ml). Increased assay error at the lower end of the standard curve may also have contributed to the observed posterior Bayesian estimates for *fu* being above a theoretical maximum of 1.0 in 8% of patients.

Similar to the study of Langenhorst et al. [12], allometric scaling using actual body weight was found to best account for the differences in body size, so our population mean estimates correspond to a subject weighing 70 kg. Sanghavi et al. conducted a study in adults (68% overweight or obese) and incorporated ideal body weight in the population pharmacokinetic model to describe F-Ara-A clearance [14]. This model, however, was not appropriate for our study since only 7 of 211 (3%) of our predominantly paediatric patient population (87%) had BMI values ≥ 30 kg/m^2^. Other studies on F-Ara-A have incorporated BSA in their models [9, 13], but current evidence supports theory-based allometry in preference to BSA [30, 37, 38]. In our study, standardisation of F-Ara-A CL and V1 to a 70 kg weight allowed us to demonstrate clear differences in these parameters across the tested age-groups, reflecting age-related changes in body composition [39], renal function [40, 41] and the expression of drug metabolising enzymes [42].

Given the importance of renal excretion in the elimination of F-Ara-A [9–15], our model for unbound F-Ara-A clearance was subdivided into non-renal and renal components, with a GFR slope factor applied to the renal component as previously described [12]. Non-renal clearance was approximately 47% in a typical patient, reflecting its cellular metabolic conversion to the 5′-triphosphate derivative, F-Ara-ATP [32], which is responsible for its cytotoxic action. Renal clearance was approximately 53% in a typical patient, consistent with previous results of 55% obtained for clofarabine, another purine nucleoside [43]. The population mean for the GFR slope factor in our model was determined to be 1.02 (95%CI: 0.83 – 1.42), suggesting that 100% of the GFR contributed to the renal excretion of F-Ara-A. This was also indicated by a lack of association between GFR and observed or model-predicted F-Ara-A in the visual predictive check with GFR as the independent variable. This is higher than the slope value of 0.78 estimated by Langenhorst et al. [12]. This difference can be accounted for by the fact that, in our study, GFR was accurately measured using a radionuclide technique [44] for the majority of paediatric patients, whilst in the Langenhorst study [12], GFR was estimated using the Cockroft-Gault and Schwartz equations. Moreover, our model estimated unbound F-Ara-A clearance and it is the unbound drug that is filtered by the kidney and undergoes renal excretion [45]. It should also be noted that the kidney also performs the physiologic functions of secretion and reabsorption in addition to filtration [45]. The fact that the population mean and upper limit of the 95%CI for the GFR slope factor was above 1.0 suggests that F-Ara-A may also be subject to secretion processes in the kidney. Active secretion of nucleoside analogues via a sodium -nucleoside transport system has been previously reported [46]. These more complex renal elimination mechanisms for F-Ara-A may also, in part, explain the altered renal maturation functions in our study when compared with that of Rhodin et al. [47], in which published data was pooled to examine GFR maturation from neonate to adults: for renal clearance our model estimated the PMA at 50% mature to be 69.1 weeks with a Hill co-efficient of 2.97 (95%CI:0.99 – 4.66), whilst Rhodin et al. obtained a population mean value at 50% mature of 47.7 weeks (95%CI 45.1 – 50.5) and a Hill co-efficient of 3.4 (95%CI: 3.03 – 3.8).

In the ADDIKD guidelines the conditional recommendations for fludarabine dosing are that the full BSA-based dose be administered for patients with GFR ≥ 60 ml/min/1.73 m^2^ (G1 and G2 categories), a 50% reduced dose be administered to patients with AML with GFR values of 30–59 ml/min/1.73m^2^ (G3A, G3B categories) and that fludarabine should not be administered when GFR < 30 ml/min/1.73m^2^ (G4 category) [18]. These recommendations are conditional since, at the time of writing (2022), there were no published studies assessing the application of KDIGO CKD categories to guide dose adjustment of fludarabine and the monitoring of adverse events, particularly in young infants and children with a variety of diagnoses. Our limited data for the G3B category (n = 2, 1 adult and 1 child) supports a 50% dose reduction based on unbound F-Ara-A AUC_0-∞_ estimates. For the G3A category, however, a 50% dose reduction would have been appropriate for only 3 (of 7, 43%) patients with three of the remaining 4 patients being within ± 14% the target AUCu_0-∞_ without dose adjustments, suggesting that target AUCu_0-∞_ intervention may be important in patients with impaired renal function. This will be facilitated by our model once it is externally validated. Alternative dosing algorithms based on allometric weight and GFR, as previously recommended [43] may also produce more consistent exposure estimates compared with BSA-based dosing, which was observed to be associated with wide variability in unbound AUC_0-∞_ estimates in this and previous [12] studies.

Clinical experience with fludarabine dosing has primarily relied on BSA-based dosing. Therefore, the target AUCu_0-∞_ values in this study were determined based on the median observed values corresponding to the relevant mg/m^2^ dose specified in the treatment protocol. Further studies are needed to identify appropriate exposure targets associated with good transplant outcome. Whilst an association between high F-Ara-A AUC_0-∞_ and lower event-free survival (Hazard ratio (HR) 2.0, 95%CI 1.1–3.5, p = 0.02, n = 192) due to increased non-relapse mortality (HR 3.4; 95%CI: 1.6–6.9, p < 0.001) has been previously identified [48], the AUC_0-∞_ determinations were based on based total F-Ara-A concentrations. To our knowledge, no pharmacodynamic studies to date have been conducted using unbound pharmacokinetic data. Our group plans to conduct such a study, which will also include an examination of F-Ara-A pharmacodynamics in neonates and infants, a group with potentially altered pharmacodynamic responses [49].

To conclude, for the first time we have developed a robust three compartment linear protein binding population pharmacokinetic model for F-Ara-A that generated estimates of unbound pharmacokinetic parameters from the combination of total and unbound concentrations. With a patient population ranging in age from neonates to adulthood, standardisation of pharmacokinetic parameter estimates to 70 kg weight, allowed us to demonstrate clear differences in unbound F-Ara-A clearance and V1 across a wide range of age groups. GFR was also identified to be an important covariate in the population pharmacokinetic model, with our F-Ara-A AUCu_0-∞_ data supporting target concentration intervention and possible dose reductions for patients in the G3A and G3B KDIGO CKD categories. Our population pharmacokinetic model for F-Ara-A generated derived pharmacokinetic parameter estimates that were similar to those obtained in a previous phase 1 trial characterised by rich data collection. The pharmacokinetic differences observed in studies utilising different structural models and sampling intensity schedules highlight a need for clinicians to be informed about the impact of these differences on model-generated dose recommendations. Furthermore, multi-disciplinary teams are needed to oversee the pharmacokinetic monitoring of these potentially life-threatening HSCT-conditioning agents to ensure that patients are delivered the optimal dose that will lead to best outcomes. Although pharmacological scientists have been studying the pharmacokinetics of agents used in transplantation for decades, clinician involvement is still relatively limited. Most clinicians understand the fundamental concepts of drug level measurement and AUC and have some knowledge of sample collection frequency and timing. However, the intricacies of using different pharmacokinetic models, that may give rise to different AUC results, may not always be apparent from a clinical perspective. Before we can extend our experience into clinical practice, we firstly have to determine the pharmacodynamic consequences of the exposure to fludarabine we have delivered, and secondly ensure that different patient’s results are correctly interpreted with respect to the modelling methods used to derive the AUC.

## Supplementary Information

Below is the link to the electronic supplementary material.

Supplementary file1 (PDF 218 KB)

## Data Availability

No datasets were generated or analysed during the current study.
