# Supplementary material for: The impact of age and renal function on the pharmacokinetics and protein binding characteristics of fludarabine in paediatric and adult patients undergoing allogeneic haematopoietic stem cell transplantation conditioning
Source: Eur J Clin Pharmacol. 2024 Sep 19;80(12):1967–87. doi: 10.1007/s00228-024-03751-0 (PMC11557628; doi:10.1007/s00228-024-03751-0)
Supplement: Supplementary file 1 — Supplementary file1 (PDF 218 KB) [file 228_2024_3751_MOESM1_ESM.pdf]

Supplementary Information: European Journal of Clinical Pharmacology

The impact of age and renal function on the pharmacokinetics and protein binding characteristics of fludarabine in children and adults undergoing allogeneic haematopoietic stem cell transplantation conditioning.

Christa E Nath[1,2,4](corresponding author, email:christa.nath@health.nsw.gov.au, orchid i.d.0000-0002-1013-3646), Sebastian PA Rosser[1,2,5] (orcid i.d. 0000-0002-0203-4904), Kiran K. Nath[6](orcid i.d. 0009-0006-7442-1555), Jason Chung[1], Stephen Larsen[3], John Gibson[3], Melissa Gabriel[2], Peter J. Shaw[2,5], Steven J. Keogh[2]

[1] Department of Biochemistry, The Children's Hospital at Westmead, Westmead, NSW, 2145, Australia,

[2] The Cancer Centre for Children, The Children's Hospital at Westmead, Westmead, NSW, 2145, Australia,

[3]Royal Prince Alfred Hospital, Camperdown, NSW, 2006, Australia

[4]Sydney Pharmacy School, University of Sydney, Camperdown, NSW, 2006, Australia

[5]The Children's Hospital at Westmead Clinical School, University of Sydney, Westmead, NSW, 2145, Australia

[6]School of Psychology, Western Sydney University, Kingswood, NSW, 2747, Australia.

Running title: Population pharmacokinetics and protein binding of fludarabine.

NONMEM control file for the three compartment linear protein binding model for F-Ara-A, the circulating metabolite of fludarabine.

=================

$PROBLEM PK Fludarabine total and unbound 3 COMPARTMENT MODEL

; =================

$INPUT

C ; Comment line
ID ; Patient identifier
INSTITUTION ; Institution flag
DAY ; Day of fludarabine dosing
TAD ; Time after dose (h)
TIME ; Time of sample collection (h)
CMT ; compartment number (1=unbound F-Ara-A, 2=total F-Ara-A)
DV ; Fludarabine concentration (mg/L)
AMT ; Fludarabine dose (mg)
RATE ; Rate of infusion (h^{-1})
IDUR ; infusion duration (h)
EVID ; Event identifier
MDV ; Missing DV
PMA ; Postmenstrual age (weeks)
AGFR ; Absolute glomerular filtration rate (mL/min)

Unused covariates

NGFR ; Normalised glomerular filtration rate (mL/min/1.73m2)
AGE ; Patient age (years)
HT ; Patient height (cm)
WT ; Patient weight (kg)
BSA ; Body surface area (m2), unused
SEX ; Patient sex, unused
DIAGNOSIS ; Patient diagnosis flag, unused
BMI ; Body-mass index (kg/m^2)
FFM ; Fat free mass (kg)
RLIVER ; existence of pre-existing liver impairment
CONDITIONING ; Conditioning agent flag
AMB ; Concomitant amphotericin B flag
FLUCONAZOLE ; Concomitant fluconazole flag
CLOB ; Concomitant clobazam flag
GANCICLOVIR ; Concomitant ganciclovir flag
CLONAZEPAM ; Concomitant clonazepam flag
ALLOPURINOL ; Concomitant allopurinol flag
ALB ; Albumin (g/L)
TPR ; Total protein (g/L)

NAME=DROP

; =================

$DATA

```
		NM_FLUTOTALUNC2.csv
		IGNORE=		C
; =================
```

$SUBROUTINE

```
	ADVAN11
	TRANS4
;=================
```

$PK

```
; NORMALISED COVARIATES
	NWT=WT/70
	NAGFR=AGFR/90

; ====================

; INITIALISE GFR
		TVGFR=NAGFR*(70/WT)

;=================

; ALLOMETRIC SCALING FACTORS
		ALLOCL=(NWT**0.75)
		ALLOV=(NWT**1.0)

;INITIALISE COVARIATE - PATIENT MATURATION on RENAL CLEARANCE
		TVPM50R = THETA(9)
		PM50R = TVPM50R
		TVHILLR = THETA(10)
		HILLR = TVHILLR
		FMATR = PMA**HILLR/(PM50R**HILLR+PMA**HILLR)

; TYPICAL VALUES FIXED EFEFCTS
		TVCLNR=THETA(1)
		TVCLR=TVGFR*THETA(2)*FMATR
		TVCL=TVCLNR+TVCLR
		TVV1=THETA(3)
		TVQ2=THETA(4)
		TVV2=THETA(5)
		TVQ3=THETA(6)
		TVV3=THETA(7)

; TYPICAL VALUES RANDOM EFFECTS
		BSVCL=ETA(1)
		BSVV1=ETA(2)
		BSVQ2=ETA(1)
```

```
                BSVV2=ETA(2)
                BSVQ3=ETA(1)
                BSVV3=ETA(2)


; LINEAR PROTEIN BINDING MODEL
TVFU = THETA(8)
BSVFU=ETA(3)
FU=TVFU*EXP(BSVFU)


; INITIALISE INTEROCCASION VARIABILITY
                DAY1    =       0
                DAY2    =       0
                DAY3    =       0
                DAY4    =       0
                DAY5    =       0
 IF(DAY.EQ.1)   DAY1    =       1
 IF(DAY.EQ.2)   DAY2    =       1
 IF(DAY.EQ.3)   DAY3    =       1
 IF(DAY.EQ.4)   DAY4    =       1
 IF(DAY.EQ.5)   DAY5    =       1

IOVCL = DAY1*EXP(ETA(4)) + DAY2*EXP(ETA(5)) + DAY3*EXP(ETA(6)) + DAY4*EXP(ETA(7)) +
DAY5*EXP(ETA(8))
IOVV = DAY1*EXP(ETA(9)) + DAY2*EXP(ETA(10)) + DAY3*EXP(ETA(11)) + DAY4*EXP(ETA(12)) +
DAY5*EXP(ETA(13)))


; PK PARAMETERS
                CL=TVCL*ALLOCL*EXP(BSVCL+IOVCL)
                V1=TVV1*ALLOV*EXP(BSVV1+IOVV)
                Q2=TVQ2*ALLOCL*EXP(BSVQ2+IOVCL)
                V2=TVV2*ALLOV*EXP(BSVV2+IOVV)
                Q3=TVQ3*ALLOCL*EXP(BSVQ3+IOVCL)
                V3=TVV3*ALLOV*EXP(BSVV3+IOVV)

; SCALING
                S1 = V1

; DISTRIBUTION
                K1O = CL/V1
                K12 = Q2/V1
                K21 = Q2/V2
                K13 = Q3/V1
                K31 = Q3/V3
                AUCU = AMT/CL
```

```
;=================
$ERROR
        (OBSERVATION ONLY)
                IPRED    =  F

; --- FRACTION UNBOUND
                FU_IJ = FU

; RESIDUAL ERROR MODEL
                CU=A(1)/V1
                CTOT = CU/FU_IJ
                AUCU_IJ=AUCU
                AUCT=AUCU/FU_IJ

                IF (CMT.EQ.1) IPRED=CU
                IF (CMT.EQ.2) IPRED=CTOT

                W = IPRED
                IF (W.EQ.0) W = 1
                IF (CMT.EQ.1) TYPE=0
                IF (CMT.EQ.2) TYPE=1
                Y = IPRED + W*EPS(1)*(1-TYPE) + W*EPS(2)*TYPE
                IRES = DV-IPRED
                IWRES = IRES/W


;=================

$THETA
                (0.1,    1.81,    100)                    ; CLNR
                (0 ,     1,       10)                     ;CLR
                (0,      9.29,    100)                    ; V1
                (0,      7.48,    100)                    ; Q2
                (0,      8.49,    100)                    ; V2
                (0,      1.51,    100)                    ; Q3
                (0,      18.5,    100)                    ; V3
                (0,      0.878,   1)                      ; FU
                (0,      69.1,    100)                    ; PM50_Renal
                 (0.5,   2.97,    5)                      ; HILL_Renal

$OMEGA  BLOCK(2)
                0.104                                     ; BSVCL ETA1
                0.05     0.162                            ; BSVV   ETA2

$OMEGA  BLOCK(1)
                 0.0188                                   ; BSVFU ETA3
```

```
$OMEGA  BLOCK(1)
                0.0176                                  ; IOVCL D1 ETA4
$OMEGA  BLOCK(1)
                SAME
$OMEGA  BLOCK(1)
                SAME
$OMEGA  BLOCK(1)
                SAME
$OMEGA  BLOCK(1)
                SAME

$OMEGA  BLOCK(1)
                 0.0307                                 ; IOVV D1 ETA9
$OMEGA  BLOCK(1)
                SAME
$OMEGA  BLOCK(1)
                SAME
$OMEGA  BLOCK(1)
                SAME
$OMEGA  BLOCK(1)
                SAME

;=================
$SIGMA
                0.0338                                  ; ERR1 UNBOUND F-ARA-A
                0.0293                                  ; ERR2 TOTAL F-ARA-A
;=================
$ESTIMATION
METHOD          =       1
INTER
MAXEVAL         =       9999
NOABORT
SIG             =       3
PRINT           =       1
POSTHOC

;=================
$COVARIANCE
;=================
$TABLE          ID TIME TAD CMT DV EVID MDV IPRED PRED IRES CWRES IWRES
                ONEHEADER NOPRINT FILE=PBfluRES.TAB
$TABLE          ID DAY CL V1 Q2 V2 Q3 V3 ETAS(1:13) TIME AUCU AUCT FU CLKG V1KG K12 K21 K13
                K31 ONEHEADER NOPRIN FILE=PBflu3CPTCOVpar002.TAB
$TABLE          ID DAY CL V1 Q2 V2 Q3 V3 AUCU AUCT FU CLKG V1KG NAGFR AGE WT MSA SEX
                DIAGNOSIS PMA BMI PFFM NGFR AGFR LIVER RLIVER CONDITIONING
                AMB FLUCONAZOLE CLOB GANCICLOVIR CLONAZEPAM ALLOPURINOL
                ALB TPR FUM ONEHEADER NOPRINT FIRSTONLY  FILE=FLUFIRST3CPTCOV001.TAB
```
